# Short and Ultra-Short Implants, in Association with Simultaneous Internal Sinus Lift in the Atrophic Posterior Maxilla: A Five-Year Retrospective Study

**DOI:** 10.3390/ma15227995

**Published:** 2022-11-12

**Authors:** Giorgio Lombardo, Annarita Signoriello, Mauro Marincola, Pietro Liboni, Paolo Faccioni, Alessandro Zangani, Antonio D’Agostino, Pier Francesco Nocini

**Affiliations:** 1 Dentistry and Maxillo-Facial Surgery Unit, Department of Surgery, Dentistry, Paediatrics and Gynaecology (DIPSCOMI), University of Verona, Piazzale L.A. Scuro 10, 37134 Verona, Italy; 2 Dental Implant Unit, Research Department, Faculty of Dentistry, University of Cartagena, Cartagena 130001, Colombia

**Keywords:** bone gain, crestal bone height, implant survival, internal sinus lift, maxilla, short implant, single crown, ultra-short implant

## Abstract

Recent short-term studies suggested the use of short and ultra-short implants in association with a modified osteotome sinus floor elevation (internal sinus lift) technique for the treatment of edentulous resorbed posterior maxilla. The aim of this retrospective study was to investigate this hypothesis in locking-taper implants with a mid-term follow-up of 5 years. Overall, 155 implants (32, 100, and 23 of, respectively, 5.0 mm, 6.0 mm, and 8.0 mm length) were positioned in the atrophic upper maxilla of 79 patients, and 151 implants were loaded with single crowns. Overall implant survival after 5 years was 94.84%. Implant survival for each length group was 93.75%, 94%, and 100% for 5.0, 6.0, and 8.0 mm length, respectively. Preoperative residual crestal bone height of 4.45 (1.3) mm increased to 9.25 (2.13) mm after implant placement and settled at 6.35 (1.73) mm after loading and at 5.25 (1.68) mm at follow-up. Elevation of the Schneiderian membrane was 4.8 (2.46) mm after implant placement, 3.06 (1.3) mm after loading, and 1.46 (1.06) mm at follow-up. Mean variations of peri-implant crestal bone loss and first bone-to-implant contact point were, respectively, −0.36 (1.3) mm and −0.62 (1.15) mm. It can be confirmed that internal sinus lift procedure revealed stable bone gain and negligible resorption at mid-term follow-up for atrophic upper crests with reduced height.

## 1. Introduction

After teeth extraction, the atrophic posterior maxilla usually encounters advanced bone resorption [1] and increased pneumatization [2], which lead to extremely reduced post-extractive residual crests. It is widely recognized that insufficient alveolar bone height or width, together with lack of adequate bone density for implant placement, affect the reliability of final prosthetic rehabilitations [3]. Regarding edentulous areas of the upper posterior maxilla, 62% of cases are represented by residual crestal bone height (RCBH) inferior to 6 mm [2]. Major surgical sinus-lift procedures [4,5,6,7,8] or even zygomatic implants [9,10] are often required in patients characterized by severe bone deficiency to obtain adequate maxillary bone volume for implant placement and successfully re-establish proper masticatory functions. In this proposal, a conventional method usually carried out for these cases of rehabilitation is the sinus floor elevation procedure with lateral antrostomy (LSFE) [4,5,11]. Depending on the available RCBH, LSFE can be implemented as one-step protocol (simultaneous implant placement with sufficient primary implant stability) or two-step protocol; in the latter case, implant placement is postponed for 6 months to allow appropriate healing of the grafting material placed into the sinus [11,12]. Although both procedures demonstrated favourable long-term implant survival and bone-levels stability [13], the one-step procedure is preferred as easier and clearly preferable for reduction of treatments and times. Nevertheless, these protocols are reported as complex and sometimes involved in post-operative complications of difficult clinical management [14,15,16].

Values of RCBH equal to 5.0 mm were estimated as acceptable to allow placement of implants with length ≥ 8.0 mm in association with sinus augmentation procedures [13]. Apart from the abovementioned procedures, the osteotome sinus floor elevation (OSFE) [17,18,19,20] technique was proposed in 1994 as a valid and conservative approach, with unquestionable intra- and post-operative advantages [21,22,23,24] in limiting morbidity, risk of infections, and overall treatment times. Although, historically, the original OSFE protocol was first reserved for placement of 10.0 mm implants in RCBH of at least 7 mm, this limit was then modified by several authors, hypothesizing the minimum RCBH that could be addressed [25,26,27,28,29]. The modified OSFE technique was finally suggested as a suitable technique even with extremely reduced RCBH ≤ 5 mm or even <4 mm. Nevertheless, survival rates seemed to significantly drop in implant sites presenting RCBH ≤ 5 mm beneath the sinus [21,22,30,31]. Furthermore, the most reported surgical complication for osteotomes procedure is the perforation of Schneiderian membrane [32,33]: as the risk of perforation is increased according to the extent of sinus floor elevation to be obtained, the ability to elevate the sinus membrane without perforation may represent a major concern in the case of highly resorbed ridges using implants with length ≥ 8.0 mm [34]. To decrease this risk in patients with RCBH ≤ 5.0 mm, recent studies proposed the use of short (≤8.0 mm) implants in association with OSFE, reporting good percentages of implant survival [35,36,37].

To the best of our knowledge, current long-term investigations on short and ultra-short implants placed in combination with the OSFE technique regard splinted implants, while evidence on the ones supporting single crowns is still scarce [38,39,40,41]. In the light of the promising findings exposed in a previously published 3-year study on the same topic [39], the authors hypothesized that short (≥6.0 mm and ≤8.0 mm) and ultra-short (<6.0 mm) implants, restored with single crowns, can represent a valid alternative for the treatment of severely reduced RCBH in the atrophic posterior maxilla even in a mid-term perspective. The aim of the study was to retrospectively evaluate the outcomes of plateau-design locking-taper implants of 8.0, 6.0, and 5.0 mm length, placed in combination with a modified osteotome sinus floor elevation procedure (ISL, internal sinus lift technique), after 5 years of follow-up.

## 2. Materials and Methods

This 5-year retrospective study was conducted according to the same methodology and criteria used in a previously published 3-year study (by the same research group) on short and ultra-short locking-taper implants placed through the ISL technique [39]. The following paragraphs and the Appendix A report materials and methods also used in this previous 3-year study on the same topic [39]. Even if some content described is equal, the present 5-year investigation constitutes a separate retrospective evaluation with its group of patients.

### 2.1. Study Design and Inclusion Criteria

Patients were recruited and treated, between January 2014 and January 2015, with implant-supported single crowns for edentulism (tooth loss caused by trauma, caries, or periodontal disease) in the posterior maxilla at the Dental and Maxillo-Facial Surgery Clinic at the University of Verona (Italy). A retrospective study with a 65-month follow-up was conducted between July and September 2020. The University Institutional Review Board approved the retrospective study (Protocol “SINUSLIFT”, 23/05/18). The nature and aim of the study, together with the anonymity in the scientific use of data, were clearly explained in a written, informative consent form, which was signed by every patient. All clinical procedures were performed in accordance with the Declaration of Helsinki and the good clinical practice guidelines for research on human beings, as previously described [39].

To be included in the study [39], patients had to have at least one 5.0 mm, 6.0 mm, or 8.0 mm length locking-taper implant, which had been placed in a partially edentulous posterior maxilla in combination with an ISL procedure and which supported a single crown. In addition, the RCBH must have been equal to or less than 6.0 mm, and the crestal bone thickness must have been of at least 6 mm, as determined by CBCT (cone beam computed tomography) scan measurements. Furthermore, the patients included presented ASA status I and II (according to the American Society of Anesthesiologists’ classification [42]), that is, respectively normal health and mild systemic diseases (without substantive functional limitations, such as current smokers, alcohol drinker, mild obesity, well-controlled diabetes mellitus, and mild lung disease). Exclusion criteria were as previously described [39] (see Appendix A).

### 2.2. Surgical Protocol

All treatments and visits were carried out by two experienced periodontal surgeons. Surgical protocol was conducted as previously described [39] (see Appendix A).

### 2.3. Prosthetic Protocol and Follow-Up Evaluation

After six months, implants were surgically uncovered, healing abutments were placed, and the mucosal flaps re-adapted and sutured around the healing abutments. After three weeks of soft tissue healing, definitive impressions were taken using a polyether material (3M ESPE Impregum Impression Material) [39]. Definitive single-crown restorations were delivered within two weeks. Considering patients’ preference, mostly guided by personal economic resources, composite or porcelain material was chosen: in the first case, a micro-hybrid composite containing 73% by weight micro-fine ceramic particles embedded in an organic polymer matrix (Ceramage, Shofu Inc., kyoto, Japan) was used; in the second case, a bilayer crown was planned using a zirconia framework veneered with feldspar ceramic (Ceramica Natural ZiR, Tressis Italia srl, Conegliano, Italy) [43].

The prosthetic technique used was the Integrated Abutment Crown (IAC): as previously described [43], single-tooth crowns are extra-orally, chemo-mechanically bonded to the coronal part of a titanium alloy non-shouldered or shouldered locking-taper abutment, and excess cement is removed. The one-piece abutment and crown are inserted into place by mean a gentle tapping, using a 250 g mallet, through a crown seating tip supplied by the manufacturer and a custom-made acrylic tapping jig to ensure accurate proper seating [43].

Regarding the occlusal scheme, the palatal contour of the implant-supported single crowns was reduced to decrease the offset load to the implant body, and the buccal cusp remained void of occlusal contact to minimize cantilever forces [44]. The occlusion was carefully monitored at the time of loading and during follow-up examinations, and occlusal adjustment were made when considered appropriate to prevent overloading; furthermore, at each recall appointment, prosthetic restorations were checked for loosening, chipping, or other types of complications.

A maintenance program was designed to provide patients a professional oral hygiene session every four months, and home care procedures were reinforced. Clinical assessment of peri-implant soft tissues and radiographic examinations were performed after five years of follow-up from loading time.

### 2.4. Implant Type Characteristics

Short (8.0 and 6.0 mm in length) or ultra-short (5.0 mm in length) implants were utilized in this study. The dental implant system (Bicon Dental Implants, Boston, MA, USA, designed in 1985) includes a locking-taper (Morse taper or Morse cone) connection, a plateaus root-form design, convergent crest module, platform switching, and an Integra CP^TM^ surface (hydroxylapatite-treated and acid-etched) [39]. The locking-taper connection supplies an impervious seal to microbial penetration or infiltration, which allows an absence of micromovements or micro gaps at the implant–abutment interface, resulting in greater mechanical stability to the implant/crown assembly and minimal bone resorption [43,45]. The plateaus design allows an increase of the implant–bone surface area, with initial woven bone formation at the healing chambers, and following haversian-like configuration significant for mechanical properties [43,46,47]. In addition, the platform design provides an implant shoulder gradually sloping inward and coronally, toward the implant–abutment interface, creating space for crestal bone, while the base of the implant abutment represents a loading surface through which compressive loads are exerted on existing or potential crestal bone [43,48]. These distinctive features in single-tooth restorations are thus extremely favourable in preserving crestal bone and consequently preventing bone loss even in the presence of unfavourable high CIR, as vertical, horizontal, and rotational forces are adequately transmitted, providing stable functioning over time [43,49].

### 2.5. Study Variables and Outcomes

Study variables and outcomes were as previously described [39] (see Appendix A). Implant lengths considered in this study were 8.0 mm, 6.0 mm, and 5.0 mm; implant diameters were 4.0 mm, 4.5 mm, and 5.0 mm. Covariates included were: sex, age, smoking history, history of periodontal disease, ASA status, number of oral hygiene sessions per year, interproximal access for oral hygiene, tooth site (premolar or molar replaced by implant), prosthetic material, crown-to-implant ratio (CIR), and pre-operative RCBH.

Patients with a history of treated periodontitis were characterized by previously assessed chronic forms of periodontal disease corresponding to stage III and grade A or B according to the latest updates on classification of periodontal and peri-implant diseases [44]. These patients were subjects following a regular maintenance program on a reduced periodontium every three months to ensure gingival health at the time of implant placement. On the other hand, periodontally healthy patients were subjects never affected by any form of periodontal disease [48,50].

The main outcome was implant survival after five years of follow-up.

The secondary outcome included variations of peri-implant bone levels and sinus floor level, as previously described [39] (see Appendix A).

A descriptive analysis of crestal bone level (CBL, average bone level around implants at mesial and distal sides, in mm) and first bone-to-implant contact (F-BIC, in mm), along with their variations ΔCBL (average bone loss) and ΔF-BIC (average apical shift of the first bone-to-implant contact point position), was conducted [39,43] (see Appendix A).

Sinus floor level (SFL) was measured on the mesial, central, and distal point of each implant as the linear distance between the IAI and the sinus floor. For each implant, at each examination interval, an average (av) mesial-distal-central value for sinus floor level (av-SFL) was calculated. The sum of av-CBL and av-SFL was calculated as the residual crestal bone height (RCBH). The vertical increase in height of the implant site (intra-sinus bone height gain, IBHG) was also calculated as the difference of the RCBH with the pre-operative RCBH to obtain the final crest height [39].

Furthermore, for a complete assessment of RCBH and intra-sinus bone height gain (IBHG), other variables were registered in detail in this 5-year follow-up study:

- Implant protrusion into the sinus (IPS), measured at implant placement as the linear distance between the sinus floor and the implant apex;
- Elevation of the Schneiderian membrane, defined as sinus lift (SL): SL was measured on the mesial, central, and distal point of each implant as the linear distance between the sinus floor and the apical point of the membrane elevation; for each implant, at each examination interval, an average (av) mesial-distal-central value for sinus floor level (av-SL) was calculated;
- Percentages of graft (β-tricalcium phosphate) resorption (GR);
- Cases of Schneiderian membrane perforation (MP).

Seven days after surgery and at the five-year follow-up examination, each patient was asked to quantify the level of their satisfaction [39], on a 1-to-10-score visual analogue scale (VAS) [51], with the implant experience (question 7 days after surgery: “Are you satisfied with your implant experience?”) and considering the potential benefits (question at 5-year follow-up: “Would undergo this type of surgery again?”).

### 2.6. Statistical Analysis

Statistical analysis was conducted as previously described [39] (see Appendix A).

## 3. Results

Description of the following results reflected the presentation scheme of the outcomes of the previous 3-year study [39].

### 3.1. Demographics

Seventy-nine patients (47 women and 32 men) were included in the retrospective study according to inclusion and exclusion criteria. Mean age at placement was 54.99 (10.23) years (range 32–77). Mean age at follow-up was 58.99 (11.6) years (range 37–79). Sixty patients (with 123 implants) had lost their teeth due to periodontal disease (in some cases, this was self-reported; in others, it was determined through patient’s dental records), while nineteen patients (with 32 implants) had lost their teeth for other reasons. Sixty-nine and ten patients were, respectively, classified with ASA status I and II. Fourteen patients were smokers: among them, eight were occasional smokers, four smoked less than five cigarettes/day, and two between five and ten cigarettes/day.

Of the 155 implants placed, 20.65% were 5.0 mm, 64.52% were 6.0 mm, and 14.84% were 8.0 mm in length. Most of the implants (72.9%) were placed in the molar area. Out of the 155 implants placed, 151 were loaded with single crowns, with 145 made of porcelain and 6 made of resin. Mean CIR was 2.13 (0.61) (range 1.31–3.64). A CIR ≥ 2 prevalence was estimated in 60.93% of the implants.

The implant distribution was analysed according to length definition (8.0, 6.0, and 5.0 mm). Significant differences regarding implant site, implant diameter, CIR, and pre-operative RCBH were found according to implant-length distribution.

The overall descriptive statistics for the study variables are presented in Table 1.

### 3.2. Implant Survival

At the uncovering stage, four implants were not osteo-integrated, and thus, four early failures (2.58%) were detected, all characterized by pre-operative RBCH inferior to 5 mm. Four implants were lost after functional loading (late failures due to excessive bone loss) in four patients at 5-year follow-up: the implant survival 65 months after loading time was thus 97.35% (147/151). The overall implant survival, considering early and late failures after 5 years of follow-up, was 94.84% (147/155). No association was found between survival and failure groups or in any of the considered covariates, as reported in Table 2.

### 3.3. Radiographic Bone Levels

Average crestal bone levels were stable between loading time and follow-up time, with a mean ∆CBL of −0.36 (1.3) mm and a mean ∆F-BIC of −0.62 (1.15) mm. Outcomes regarding CBL, F-BIC, RCBH, IBHG, IPS, SL, and GR at each time interval are listed in Table 3. Even if statistically significant differences between time intervals were found, we can assume these variations as not clinically relevant: average values obtained for CBL, F-BIC, RCBH, IBHG, IPS, SL, and GR after five years of follow-up are compatible with clinical bone levels’ stability (Table 3).

As implant length, pre-operative RCBH, and history of periodontal disease were considered clinically relevant covariates, the comparisons for CBL, F-BIC, RCBH, IBHG, IPS, SL, and GR between implant length groups, RCBH groups, and perio-groups are reported in Table 4, Table 5 and Table 6.

Regarding membrane perforations (MP), 12 cases (7.74%) were totally registered. Implant survival dropped to 83.33% (10/12) in the case of MP compared to a percentage of 95.8% (137/143) in cases of non-MP, without significant differences between groups. Table 7 reports MP according to implant length, values of pre-operative RBCH, and history of periodontal disease. Furthermore, along with the years of surgical practice, percentages of perforations decreased from 50% (0–3 years) to 33% (3–5 years) and finally to 17% (>5 years of practice).

### 3.4. Patients’ Level of Satisfaction

Seven days following the surgery, when questioned about their level of satisfaction with the implant procedures, 40 patients (50.63%) gave a score between 9 and 10, 27 (34.18%) gave a score between 7 and 8, and 12 (15.19%) gave a score between 5 and 6; furthermore, five patients referred to “being hammered”, and two reported “difficult in bearing”. At five-year recall appointment, when asked the same question and whether they would undergo the surgery again, none of them retained a negative memory of the entire procedure. On the contrary, they all said that they would undergo the treatment again, and the average score was higher compared to the average score related to seven days after surgery. More precisely, 55 patients (69.62%) gave finally a score between 9 and 10, and 24 (30.38%) gave a score between 6 and 8. Statistically significant differences (*p* < 0.001) were found between the first and second time of evaluation (Table 8).

The following Table 9 summarizes main findings related to comparisons between the 3-year previously published study [39] and the 5-year present study; moreover, main findings of the 3-year intermediate follow-up of the present study are also reported.

This 5-year study (see Table 9) considered a bigger number of implants and patients (155/79 vs. 51/31). Regarding overall implant survival, data were comparable after 3 years (96.08% and 97.42%), with a small decrease after 5 years (94.84%). Bone-level and sinus-level variations remained stable from loading to 3-year follow-up for the 3-year study and from loading to 3-year and finally to 5-year follow up for the 5-year study. First bone-to-implant contact was stable over time in both studies, with similar outcomes at follow-up. Even if average values of residual crest in the 5-year study resulted a bit lower compared to the 3-year study, the general trend of values over time was comparable between the two studies, with similar decreasing variations. The same trend as residual crest can be observed for sinus lift. Satisfaction scores were high in both studies.

**Figure 1:**
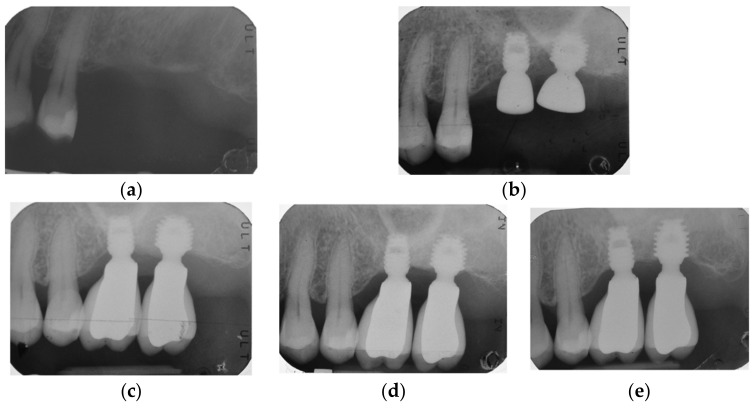
Single implants placed in 2.6 and 2.7 sites (4.5 × 6 mm and 5 × 6 mm): (**a**) pre-operative radiograph before implant placement; see minimal bone levels (2.6 RCBH = 4.5 mm; 2.7 RCBH = 4.7 mm); (**b**) radiograph obtained at time of placement; see augmented sinus floor (2.6 sinus lift = 2.3 mm; 2.7 sinus lift = 2 mm); (**c**) radiograph obtained at time of loading; (**d**) radiograph obtained at 3-year follow-up; (**e**) radiograph obtained at 5-year follow-up. See stable bone levels with minimal changes between 3-year (2.6 RCBH = 5.2 mm; 2.6 sinus lift = 1 mm; 2.7 RCBH = 5.55 mm; 2.7 sinus lift = 1.3 mm) and 5-year follow-up (2.6 RCBH = 5 mm; 2.6 sinus lift = 1 mm; 2.7 RCBH = 5.35 mm; 2.7 sinus lift = 1.15 mm).

**Figure 2:**
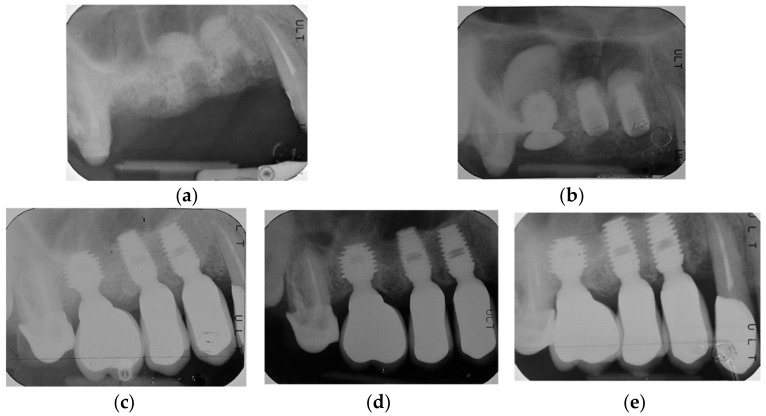
Single implants placed in 1.4, 1.5, and 1.6 sites (4.5 × 8 mm, 4.5 × 8 mm and 5 × 6 mm): (**a**) pre-operative radiograph before implant placement; (**b**) radiograph obtained at time of placement; see implant at 1.6 site with sinus lift temporary abutment designed to prevent displacement of the implant into the sinus, also see augmented sinus floor (1.4 sinus lift = 3.8 mm; 1.5 sinus lift = 2 mm; 1.6 sinus lift = 8.5 mm); (**c**) radiograph obtained at time of loading; (**d**) radiograph obtained at 3-year follow-up; (**e**) radiograph obtained at 5-year follow-up. See graft resorption after 3 years and stable bone levels with minimal changes between 3-year (1.4 RCBH = 7.5 mm; 1.4 sinus lift = 2.55 mm; 1.5 RCBH = 6.45 mm; 1.5 sinus lift = 0.5 mm; 1.6 RCBH = 7.1 mm; 1.6 sinus lift = 4.5 mm) and 5-year follow-up (1.4 RCBH = 7.12 mm; 1.4 sinus lift = 2.17 mm; 1.5 RCBH = 6.17 mm; 1.5 sinus lift = 0.47 mm; 1.6 RCBH = 6.45 mm; 1.6 sinus lift = 4.2 mm).

**Figure 3:**
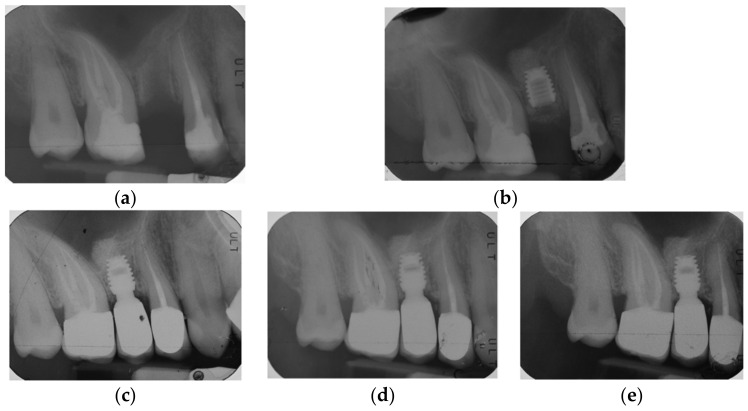
Single implant placed in 1.5 site (4.5 × 6 mm): (**a**) pre-operative radiograph before implant placement; (**b**) radiograph obtained at time of placement; see augmented sinus floor (sinus lift = 3.33 mm); (**c**) radiograph obtained at time of loading; (**d**) radiograph obtained at 3-year follow-up; (**e**) Radiograph obtained at 5-year follow-up. See minimal graft resorption after 3 years and stable bone levels with minimal changes between 3-year (RCBH = 6.5 mm; sinus lift = 3.1 mm) and 5-year follow-up (RCBH = 6.2 mm; sinus lift = 2.8 mm).

**Figure 4:**
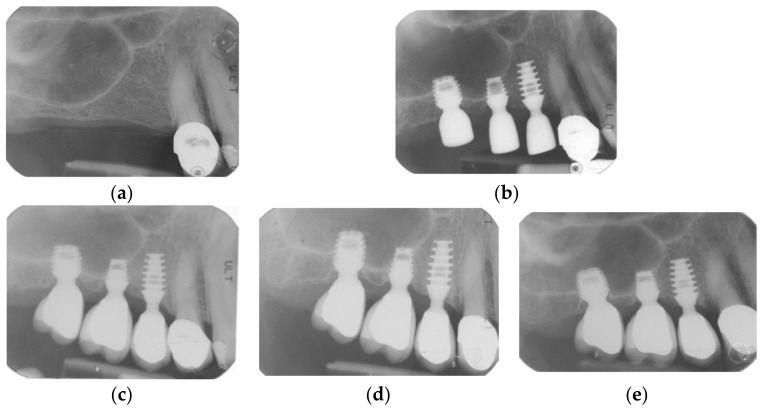
Single implants placed in 1.5, 1.6, and 1.7 sites (4 × 6 mm, 4 × 5 mm and 4.5 × 5 mm): (**a**) pre-operative radiograph before implant placement; see minimal bone levels (1.5 RCBH = 5.65 mm; 1.6 RCBH 4.2 mm; 1.7 RCBH = 3.5 mm); (**b**) Radiograph obtained at time of placement; see augmented sinus floor (1.5 sinus lift = 2 mm; 1.6 sinus lift = 2.2 mm; 1.7 sinus lift = 2 mm); (**c**) radiograph obtained at time of loading; see clear protrusion of two implants into the sinus; (**d**) radiograph obtained at 3-year follow-up; (**e**) radiograph obtained at 5-year follow-up. See large graft resorption after 3 years: the material immediately placed can be seen only with great magnification; it is then completely resorbed; however, it can be observed that finally the new necessary functional bone has been formed. However, see stable bone levels with minimal changes between 3-year (1.5 RCBH = 5.8 mm; 1.5 sinus lift = 2 mm; 1.6 RCBH = 4.2 mm; 1.6 sinus lift = 2 mm; 1.7 RCBH = 2.7 mm; 1.7 sinus lift = 0.5 mm) and 5-year follow-up (1.5 RCBH = 5.5 mm; 1.5 sinus lift = 1.8 mm; 1.6 RCBH = 3.8 mm; 1.6 sinus lift = 1.5 mm; 1.7 RCBH = 2.5 mm; 1.7 sinus lift = 0.5 mm).

By way of illustration, Figure 1, Figure 2, Figure 3 and Figure 4 report some radiographic cases with 5-year follow-up.

## 4. Discussion

Compared to standard-length implants placement in association with augmentation procedures [52,53], the use of short implants seems to offer simplified surgical protocols, with considerable enhancement of patient comfort. Nevertheless, there is no current agreement on short implants’ definition: some authors give a length definition of less than 10 mm [54], whereas others cite one inferior or equal to 8 mm [55,56,57]. The present study considered, respectively, 8.0- and 6.0-mm length implants as short and 5 mm length implants as ultra-short [43].

The option offered by these reduced-length implants, in the case of RCBH inferior to 5 mm, is unavoidably often associated with the OSFE procedure: in a single surgical session, using tapered osteotomes with increasing diameters, an osteotomy is created for implant placement, and by gently tapping the osteotome in a vertical direction, a fracture is performed in the maxillary sinus floor, and the membrane is lifted, thus creating a space that can be grafted with different materials prior to implant placement [17,18,19,20,21]. The pre-treatment of atrophic residual crest still represents a major issue when discussing the indications for OSFE procedure in association with standard implants or even with short or ultra-short implants. A systematic review by Del Fabbro et al. [58] on 3131 implants placed with sinus elevation via a crestal approach found an implant survival of 96.9% and 92.7%, respectively, for RCBH of more than 5 mm and less than 5 mm; in this study, the minimum value for RCBH compatible with acceptable results was set at 5 mm. On the contrary, some investigations assessed higher percentages of implant survival for RCBH inferior to 5 mm, with implants of at least 8 mm length, placed with the OSFE technique: Bernardello et al. [59] showed an implant survival of 96.3% after 4 years of follow-up; Bruschi et al. [60] reported a percentage of 95.8% after 5 years for RCBH ≤ 3 mm; French et al. [61] obtained values of 98.3% after 5 years of follow-up. Other authors [23,62,63] highly recommended a delayed implant placement to reach adequate bone-level stability and prevent risk of failures. A recent meta-analysis [64] showed that short implants in association with OSFE technique revealed equal or even superior results compared to standard implants associated with LSFE and bone grafting for patients with intermediate maxillary RCBH (4–8 mm); moreover, it was also suggested that LSFE does not represent anymore a suitable therapeutic option because of unjustified increase of complications and financial costs.

Concerning prosthetic aspects, it is currently debated whether short and ultra-short implants placed in resorbed alveolar ridges may be rehabilitated with single crowns, which usually allow easier hygiene procedures, have a passively fitting framework, and typically demonstrate better aesthetics [43]. Nevertheless, several recent systematic reviews [65,66,67,68] with 5 years of follow-up recommended that short implants should be splinted whenever possible. According to this, some authors described favourable results for splinted short implants in the atrophic posterior maxilla placed in native bone with RCBH greater than 5 mm and in combination with OSFE procedure in cases of RCBH < 5 mm [35,36,37]. On the other hand, most of the studies on short and ultra-short implants rehabilitated with single crowns and with a follow-up of at least 5 years did not report promising outcomes in terms of implant survival and bone levels [69,70,71]. To the best of our knowledge, only few studies characterized by short-term evaluations presented outcomes of short and ultra-short implants placed in the atrophic posterior maxilla in association with the OSFE protocol and supporting single crowns [39,40,41]. Specific factors mostly related to the relationship between implant design, augmented CIR, and marginal bone loss may play a fundamental role in influencing results. For most of the screw-root form implant macro-designs available, in presence of high lateral masticatory forces, a single crown with increased CIR can usually determine excessive marginal bone loss, which finally leads to implant failure [72,73]. Differently, in a recent study [43] of the same research group on locking-taper short and ultra-short implants with a 5-year follow-up, thirty-nine 5.0 mm and forty-one 6.0 mm length implants, supporting single crowns in the posterior resorbed maxilla, offered even in presence of augmented CIR stable outcomes in terms of implant survival and bone-level stability, not statistically different from those obtained by fifty-six 8.0 mm length implants with the same design.

Outcomes of the present 5-year follow-up, again, even in presence of unfavourable high CIR, may be explained by: (i) the specific implant macro-design with plateaus, which increases the implant–bone surface area when compared to implants of similar dimensions but with screw-root form macro-design [43] and (ii) the impervious seal conferred by the locking-taper implant-abutment connection [46,74]. A study by Chou et al. [46] reported bone density distributions such as natural tooth, which lead the authors to conclude that plateau-design implants are more suitable in preventing bone loss.

Promising results in terms of increased unfavourable CIR and reduced RBCH were already reported by previous short-term studies [39,40,41,75] on short and ultra-short locking-taper implants placed in the atrophic posterior maxilla in association with the OSFE protocol and supporting single crowns. Nizam et al. [40] evaluated 29 short and ultra-short locking-taper implants placed in conjunction with osteotome sinus floor elevation and rehabilitated with single crowns, showing after 2 years an overall survival rate of 93.1% (two failures were detected before loading) and a survival rate after loading of 100%. Lombardo et al. [39] followed for three years 21 ultra-short and 23 short implants placed in combination with a modified OSFE procedure and presenting a mean CIR of 1.99, finding after three years an implant survival of 95.4%; no failures were detected before loading and two failures took place after loading, with an overall implant survival and an implant survival after loading, respectively, of 100% and 95.4%. A recent retrospective study by Carelli et al. [41] on 102 patients reported a three-dimensional investigation at one year and five years of follow-up for 26 patients, finding no failures for 30 trans-crestal sinus floor elevations with immediate implant placement in the severely atrophic maxilla.

Our outcomes provided an overall implant survival of 94.84% (147/155): 93.75%, 94%, and 100% for 5.0, 6.0-, and 8.0-mm length groups, respectively. Four of the eight total failures were early failures (2.58% out of 155 placed implants, in line with other authors [76]), with two in 5.0 mm group and two in 6.0 mm length group: this issue may be of clinical relevance since it could be related to the scarce primary stability offered by a short or ultra-short implant placed in a limited and poor-quality residual bone. However, four implants failed after loading in 5.0 mm and 6.0 mm groups, while no failures were registered for 8.0 mm group (implant survival after 5 years of loading was 97.35% (147/151)). Furthermore, 5.0 mm and 6.0 mm length implants were placed in RCBH inferior to 5 mm (4.4 mm) and presented a CIR greater than 2 (respectively, 2.45 and 2.11), while 8.0 mm length implants were placed in RCBH of at least 5 mm and presented a CIR of 1.6. Although authors of the present study prudently recommend pre-operative RCBH of 5 mm to achieve adequate implant stability and osseointegration, short and ultra-short implants associated with OSFE technique finally were shown to represent a reliable option of treatment, even in case of RCBH < 5 mm, if provided a minimal and clinically negligible bone resorption between loading and follow-up time, not statistically different from the one presented by longer implants.

As regards mean IBHG after 5 years, the implant seemed to exert a “support pole” function, meaning that the membrane, initially elevated, then goes down to recline to the implant itself; the implant thus appears to often protrude into the sinus. The limited increment of RCBH values after 5 years is related to the β-TCP (β-tricalcium phosphate) material used as grafting material: pure-phase β-TCP reduces its volumetric mass at the same rate as new bone forms, and it is fully resorbed and replaced by vital bone over 6 months, as shown histologically in animal studies, whereas bovine-derived grafts are not [77,78]. From these studies [77,78], we know that the mean resorption rate of β-TCP graft is around 80% and that β-TCP graft cannot fulfil a function as a space maintainer; however, its replacement ensures the fundamental regeneration of a bone that will be able to remodel according to the stresses placed upon it in the future [78]. At this proposal, patients with history of periodontal disease (79.35% of the entire sample) showed a greater tendence in GR at 5-year follow-up. The specific property of β-TCP graft seems particularly relevant for these patients: outcomes regarding bone-level changes over time were finally stable and did not statistically differ from healthy patients, underlying the feasibility of short implants rehabilitations in atrophic crests previously treated for chronic forms of periodontal disease.

Perforation of the Schneiderian membrane (MP) represents the most common complication during sinus elevation procedures, and its incidence can vary from 6.5% to 60%, depending on the authors [79,80]. Some endoscopic studies illustrated this risk even when trans-alveolar sinus floor elevation is performed. According to reports in the literature on membrane perforations with OSFE, the risk increases when the sinus membrane is lifted more than 3.0 mm [33,81]. Moreover, a thin membrane, in conjunction with septa, was related to an increased risk of membrane perforation [82]. In the present study, 12 cases (7.74%) of MP were totally registered, and other authors reported these percentages of MP: 4.7% for Toffler [40], 16% for Nedir [83], and 10.4% for Pjetrusson [13]. Differences concerning implant survival in MP and non-MP, although not statistically significant, can be assumed as clinically relevant. Pre-operative RCBH less than 4–5 mm presented a higher prevalence of membrane perforation, probably due to the major entity of membrane elevation in these groups. Furthermore, along with the years of surgical practice, the decrease in percentages of perforations seems to give evidence to the fact that the technique of placing short and ultra-short implants in association with OSFE procedure requires a gradual learning process.

Finally, the ISL technique revealed to be highly accepted by patients, as described in five-year outcomes regarding patients’ levels of satisfaction.

As in the previous 3-year investigation [39], some critical issues related to the retrospective nature of the study remain: a small sample size, a non-homogeneous distribution among implant length-groups and perio-groups, a single centre (the University Dental Clinic), and difficulties in performing follow-up appointments during the pandemic time. Even if most of the patients enrolled in the study were characterized by a history of periodontal disease, it did not negatively influence bone-levels stability over time. Compared to the previous 3-year study, a follow-up of 5 years should be considered as valid, together with a more accurate analysis of bone variations in different time intervals and a better assessment of variables related to post-operative complications (MP). Further long-term investigations (more than 5 years) with a prospective approach and a larger sample size are needed to corroborate our results in the atrophic posterior maxilla.

## 5. Conclusions

Within the limits of the present mid-term 5-year evaluation, our clinical and radiographic outcomes suggested short and ultra-short locking-taper implants, placed in conjunction with an ISL technique and restored with single crowns, as a predictable treatment for edentulous posterior maxillary regions with RCBH even less than 5.0 mm.

## Figures and Tables

**Table 1:** Overall characteristics of 155 implants placed and 151 implants loaded. Length group distribution according to study variables. Age at follow-up, months at follow-up time (from loading time), and oral professional hygiene/year are presented as median (iqr); for all other variables, values are presented as n (%); iqr, interquartile range.

VARIABLE	Overall (N = 155 Placed; 151 Loaded)		5 mm (N = 32 Placed; 30 Loaded)		6 mm (N = 100 Placed; 98 Loaded)		8 mm (N = 23 Placed and Loaded)		*p*-Value
	**n**	**%**	**n**	**%**	**n**	**%**	**n**	**%**	
SEX									
Male	74	47.74	13	40.62	51	51	10	43.48	0.53
Female	81	52.26	19	59.38	49	49	13	56.52	
AGE AT FOLLOW-UP	58.99 (11.6)		57.63 (15.04)		62.46 (10.60)		58.34 (18.57)		0.68
MONTHS AT FOLLOW-UP TIME	65 (50)		64.5 (68.5)		66 (48.5)		65 (44)		0.28
SMOKING									
No	126	81.29	25	78.12	82	82	19	82.61	0.87
Yes	29	18.71	7	21.88	18	18	4	17.39	
ASA STATUS									
I	131	84.52	24	75	89	89	18	78.26	0.1
II	24	15.48	8	25	11	11	5	21.74	
ORAL HYGIENE SESSIONS/ YEAR	3 (2)		3 (2)		3 (2)		3 (1)		0.49
INTERPROXIMAL ORAL HYGIENE									
No	44	28.39	11	34.38	25	25	8	34.78	0.45
Yes	111	71.61	21	65.62	75	75	15	65.22	
HISTORY OF PERIODONTAL DISEASE									
No	32	20.65	8	25	21	21	3	13.04	0.56
Yes	123	79.35	24	75	79	79	20	86.96	
TYPE OF TOOTH REPLACED									
Premolar	42	27.10	8	25	20	20	14	60.87	<0.001
Molar	113	72.90	24	75	80	80	9	39.13	
IMPLANT DIAMETER									
4 mm	12	7.75	2	6.25	3	3	7	30.43	<0.001
4.5 mm	51	32.90	0	0	36	36	15	65.22	
5 mm	92	59.35	30	93.75	61	61	1	4.35	
PROSTHETIC MATERIAL									
Resin	6	3.97	3	10	3	3.06	0	0	0.06
Porcelain	145	96.03	27	90	95	96.94	23	100	
CROWN LENGTH	12.6 (2.7)		12.25 (3)		12.7 (2.7)		12.8 (2.4)		0.31
CROWN-TO-IMPLANT RATIO	2.13 (0.61)		2.45 (0.6)		2.11 (0.45)		1.6 (0.3)		<0.001
CROWN-TO-IMPLANT RATIO									
<2	59	39.07	4	13.34	35	35.72	20	86.96	<0.001
2–2.99	85	56.29	22	73.33	60	61.22	3	13.04	
>2.99	7	4.64	4	13.33	3	3.06	0	0	
PRE-OPERATIVE RCBH	4.45 (1.3)		4.45 (1.2)		4.4 (1.23)		5.25 (1.05)		<0.001
PRE-OPERATIVE RCBH									
<4 mm	41	26.45	10	31.25	30	30	1	4.35	<0.001
4–5 mm	70	45.16	17	53.12	45	45	8	34.78	
>5 mm	44	28.39	5	15.63	25	25	14	60.87	

**Table 2:** Analysis of implant survival on 155 implants placed and 151 loaded implants according to included study covariates. For all variables, values are presented as n (%).

VARIABLE	Implant Survival		Implant Failure		*p*-Value
	n	%	n	%	
SEX					
Male	72	97.30	2	2.70	0.28
Female	75	92.59	6	7.41	
SMOKING					
No	119	94.44	7	5.56	0.53
Yes	28	96.55	1	3.45	
ASA STATUS					
I	123	93.89	8	6.11	0.61
II	24	100.00	0	0.00	
ORAL HYGIENE SESSIONS/ YEAR	3 (1)		2.5 (1.5)		0.19
INTERPROXIMAL ORAL HYGIENE					
No	41	93.18	3	6.82	0.68
Yes	106	95.50	5	4.50	
HISTORY OF PERIODONTAL DISEASE					
No	29	90.63	3	9.38	0.36
Yes	118	95.93	5	4.07	
IMPLANT LENGTH					
5 mm	30	93.75	2	6.25	0.65
6 mm	94	94.00	6	6.00	
8 mm	23	100.00	0	0.00	
IMPLANT DIAMETER					0.34
4 mm	11	91.67	1	8.33	
4.5 mm	50	98.04	1	1.96	
5 mm	86	93.48	6	6.52	
TYPE OF TOOTH REPLACED					
Premolar	41	97.62	1	2.38	0.68
Molar	106	93.81	7	6.19	
PROSTHETIC MATERIAL					
Resin	6	100.00	0	0.00	0.84
Porcelain	141	97.24	4	2.76	
CROWN-TO- IMPLANT RATIO					
<2	57	96.61	2	3.39	0.84
2–2.99	83	97.65	2	2.35	
>2.99	7	100.00	0	0.00	
PRE-OPERATIVE RCBH					
<4 mm	38	92.68	3	7.32	
4–5 mm	66	94.29	4	5.71	0.52
>5 mm	43	97.73	1	2.27	

**Table 3:** RCBH (residual crestal bone height), IBHG (intra-sinus bone height gain), IPS (implant protrusion into the sinus), SL (sinus lift), GR (graft resorption), CBL (crestal bone level), and F-BIC (first bone-to-implant contact point). Values for RCBH, IBHG, IPS, SL, CBL, and F-BIC are presented as median (iqr) and (max; min) at each time interval; iqr, interquartile range. Values for GR are presented as n (%).

	PRE- OPERATIVE	AFTER IMPLANT PLACEMENT	*p*-Value	AFTER LOADING	*p*-Value	AT 5-YEAR FOLLOW-UP	*p*-Value
RCBH	4.45 (1.3) (0.56; 6.2)	9.25 (2.13) (3.91; 14.55)	<0.001	6.35 (1.73) (−0.06; 9.87)	<0.001	5.25 (1.68) (−2.15; 9.46)	<0.001
IBHG		2.4 (1.45) (−0.85; 6.5)		0.56 (1.13) (−2.76; 3.66)	<0.001	−0.92 (0.99) (−3.65; 1.5)	<0.001
IPS		2.4 (1.7) (0.2; 6.8)		3.06 (1.3) (0.4; 6.15)	<0.001	1.46 (1.06) (0.03; 4.82)	<0.001
SL		4.8 (2.46) (1.9; 9.7)		3.06 (1.3) (0.4; 6.15)	<0.001	1.46 (1.06) (0.03; 4.82)	<0.001
GR		2 (1.32)		38 (25.17)		127 (86.39)	<0.001
CBL		1.6 (0.9) (−0.55; 6.75)		0.35 (0.4) (−0.45; 2.39)	<0.001	0.9 (1.08) (−2.35; 2.4)	<0.001
F-BIC				1 (0.74) (−1.08; 2.95)		0.5 (0.65) (−1.35; 3.4)	<0.001

**Table 4:** Comparison of RCBH, IBHG, IPS, SL, GR, CBL, and F-BIC at each time interval and between implant length groups. Values for RCBH, IBHG, IPS, SL, CBL, and F-BIC are presented as median (iqr) and (max; min) at each time interval; iqr, interquartile range. Values for GR are presented as n (%).

IMPLANT LENGTH	5 mm	6 mm	8 mm	*p*-Value
RCBH				
Pre-operative	4.45 (1.2) (0.56; 5.7)	4.4 (1.23) (0.75; 5.9)	5.25 (1.05) (3.2; 6.2)	<0.001
After implant placement	8.54 (1.86) (3.91; 13.23)	9.46 (2.05) (6.48; 14.55)	9.81 (2.35) (7.26; 14.31)	0.002
After loading	5.57 (1.66) (2.77; 7.63)	6.44 (1.48) (−0.06; 9.87)	6.85 (2.5) (4.05; 9.15)	0.001
At 5-year follow-up	4.94 (1.73) (1.31; 7.48)	5.08 (1.61) (−2.1; 7.76)	5.71 (2.15) (2.55; 9.46)	0.01
IBHG				
After implant placement	1.88 (1.5) (−0.85; 5.53)	2.56 (1.6) (−0.65; 6.5)	2.06 (1.3) (1.06; 4.3)	0.06
After loading	0.36 (0.94) (−2.76; 2.03)	0.67 (0.98) (−1.3; 3.66)	−0.91 (0.95) (−3.48; 1.5)	0.20
At 5-year follow-up	−0.92 (0.93) (−3.65; 0.6)	−0.91 (0.95) (−3.48; 1.5)	−0.98 (1.22) (−3.5; 1.33)	0.95
IPS				
After implant placement	2.4 (1.6) (0.6; 5.53)	2.3 (1.7) (0.5; 6.38)	2.6 (1.8) (0.2; 6.8)	0.5
After loading	2.96 (1.43) (1.06; 5.43)	3.05 (1.2) (0.4: 6.15)	3.4 (2) (1.45; 4.9)	0.46
At 5-year follow-up	1.25 (1) (0.43; 4.34)	1.48 (1.06) (0.03; 4.82)	1.56 (2.13) (0.3; 4.060)	0.51
SL				
After implant placement	4.5 (2.3) (1.9; 8.03)	5 (2.28) (2.26; 9.7)	4.8 (2.43) (2.8; 8.66)	0.17
After loading	2.96 (1.43) (1.06; 5.43)	3.05 (1.2) (0.4; 6.15)	3.4 (2) (1.45; 4.9)	0.46
At 5-year follow-up	1.25 (1) (0.43; 4.34)	1.48 (1.06) (0.03; 4.82)	1.56 (2.13) (0.3; 4.06)	0.51
GR				
After implant placement	1 (3.33)	1 (1.02)	0 (0.00)	0.58
After loading	9 (30.00)	23 (23.47)	6 (26.09)	0.76
At 5-year follow-up	27 (90.00)	81 (86.17)	19 (82.61)	0.68
CBL				
After implant placement	1.75 (1.02) (0.5; 3.35)	1.5 (0.87) (0.33; 4.45)	1.7 (0.75) (−0.55; 6.75)	0.21
After loading	0.3 (0.45) (0.01; 2.39)	0.4 (0.45) (−0.45; 2.05)	0.9 (1.4) (−1.5; 2.2)	0.23
At 5-year follow-up	0.95 (0.85) (−1.4; 2.4)	0.9 (1.08) (−2.35; 2.1)	0.9 (1.4) (−1.5; 2.2)	0.78
F-BIC				
After loading	1.12 (0.67) (−0.3; 2.1)	0.94 (0.75) (−1.08; 2.35)	1.11 (0.78) (−0.3; 2.95)	0.38
At 5-year follow-up	0.55 (0.65) (0.01; 2.45)	0.45 (0.6) (−1.35; 3.4)	0.5 (0.8) (0.15; 1.8)	0.50

**Table 5:** Comparison of RCBH, IBHG, IPS, SL, GR, CBL, and F-BIC at each time interval and between pre-operative RCBH groups. Values for RCBH, IBHG, IPS, SL, CBL, and F-BIC are presented as median (iqr) and (max; min) at each time interval; iqr, interquartile range. Values for GR are presented as n (%).

PRE-OPERATIVE RCBH	<4 mm	4–5 mm	>5 mm	*p*-Value
RCBH				
Pre-operative	3.3 (0.9) (0.56; 3.85)	4.45 (0.4) (4; 5)	5.25 (0.42) (5.05; 6.2)	0.87
After implant placement	8.8 (2.33) (3.91; 11.35)	9.36 (2.1) (6.18; 14.55)	9.8 (1.94) (7.31; 14.31)	0.97
After loading	5.88 (1.68) (−0.06; 8.5)	6.33 (1.35) (1.46; 9.87)	6.7 (1.99) (3.8; 9.86)	0.87
At 5-year follow-up	4.36 (2.13) (−2.15; 7.76)	5.14 (1.67) (1.48; 7.73)	5.65 (1.36) (2.55; 9.46)	0.73
IBHG				
After implant placement	2.16 (1.33) (−0.85; 4.3)	2.41 (1.7) (−0.65; 6.5)	2.38 (1.41) (0.73; 5.56)	0.82
After loading	0.2 (1.26) (−2.76; 2.34)	0.6 (1.03) (−1.86; 2.83)	0.61 (1.18) (−2; 3.66)	0.67
At 5-year follow-up	−1.37 (1.6) (−3.65; 0.14)	−0.78 (0.96) (−3.23; 1.5)	−0.73 (1.3) (−3.5; 1.1)	0.51
IPS				
After implant placement	3.7 (1.9) (0.8; 6.38)	2.3 (1.15) (0.7; 6)	1.85 (1.1) (0.2; 6.8)	0.82
After loading	3.53 (1.5) (1.53; 5.82)	2.76 (1.08) (0.56; 6.15)	1.85 (1.1) (0.2; 6.8)	0.67
At 5-year follow-up	1.66 (1.73) (0.03; 4.82)	1.53 (1.06) (0.3; 4)	1.2 (0.93) (0.2; 4.06)	0.51
SL				
After implant placement	5.76 (2.28) (2.7; 8.71)	4.83 (2.2) (1.9; 9.7)	4.46 (2) (1.93; 8.66)	0.82
After loading	3.53 (1.5) (1.53; 5.82)	2.76 (1.08) (0.56; 6.15)	2.76 (1.46) (0.4; 4.96)	0.67
At 5-year follow-up	1.66 (1.73) (0.03; 4.82)	1.53 (1.06) (0.3; 4)	1.2 (0.93) (0.2; 4.06)	0.51
GR				
After implant placement	1 (2.56)	1 (1.47)	0 (0.00)	0.73
After loading	16 (41.03)	15 (22.06)	7 (15.91)	0.06
At 5-year follow-up	35 (92.11)	59 (89.39)	33 (76.74)	0.1
CBL				
After implant placement	1.55 (1) (0.33; 4.45)	1.5 (0.95) (−0.55; 4.3)	1.72 (0.65) (0.51; 6.75)	0.21
After loading	0.4 (0.8) (−0.45; 2.39)	0.32 (0.37) (0.01; 1.35)	0.35 (0.27) (0.05; 1.25)	0.23
At 5-year follow-up	0.77 (1.15) (−1.4; 2.39)	0.9 (1.15) (−2.35; 2)	0.9 (0.7) (−1.5; 2.4)	0.78
F-BIC				
After loading	1 (1.4) (−1.08; 2.45)	1.25 (0.91) (−0.75; 2.35)	0.95 (0.92) (−1; 2.95)	0.38
At 5-year follow-up	0.4 (0.6) (−1.08; 2.45)	0.55 (0.6) (−1.35; 3.4)	0.45 (0.7) (−0.77; 1.8)	0.50

**Table 6:** Comparison of RCBH, IBHG, IPS, SL, GR, CBL, and F-BIC at each time interval and between perio-groups. Values for RCBH, IBHG, IPS, SL, CBL, and F-BIC are presented as median (iqr) and (max; min) at each time interval; iqr, interquartile range. Values for GR are presented as n (%).

HISTORY OF PERIODONTAL DISEASE	NO	YES	*p*-Value
RCBH			
Pre-operative	3.02 (1.22) (0.65; 5)	2.8 (1.5) (−3.7; 5.5)	0.97
After implant placement	9.13 (1.51) (6.18; 13.4)	9.35 (2.15) (3.91; 14.55)	0.93
After loading	6.58 (1.93) (1.46; 9.86)	6.33 (1.53) (−0.06; 9.87)	0.47
At 5-year follow-up	5.31 (1.67) (1.46; 7.76)	5.08 (1.65) (−2.15; 9.46)	0.64
IBHG			
After implant placement	2.43 (1.5) (0.33; 5.4)	2.38 (1.48) (−0.85; 6.5)	0.81
After loading	0.8 (1.6) (−1.3; 3.66)	0.53 (1.03) (−2.76; 2.83)	0.31
At 5-year follow-up	−0.8 (0.9) (−3.03; 1.01)	−0.85 (1.22) (−3.65; 1.5)	0.29
IPS			
After implant placement	2.3 (1.9) (0.6; 4.2)	2.4 (1.6) (0.2; 6.8)	0.38
After loading	3.1 (1.1) (0.4; 5.6)	3.05 (1.43) (1.06; 6.15)	0.92
At 5-year follow-up	1.56 (1.03) (0.2; 3.73)	1.45 (1.06) (0.03; 4.82)	0.76
SL			
After implant placement	4.36 (1.76) (2.03; 8.8)	5 (2.46) (1.9; 9.7)	0.39
After loading	3.1 (1.1) (0.4; 5.6)	3.05 (1.43) (1.06; 6.15)	0.92
At 5-year follow-up	1.56 (1.03) (0.2; 3.73)	1.45 (1.06) (0.03; 4.82)	0.77
GR			
After implant placement	0 (0.00)	2 (1.67)	0.63
After loading	8 (25.81)	30 (25)	0.92
At 5-year follow-up	22 (75.86)	105 (88.98)	0.07
CBL			
After implant placement	1.7 (0.82) (0.4; 4.3)	1.55 (0.99) (−0.55; 6.75)	0.70
After loading	0.35 (0.3) (0.05; 1.35)	0.35 (0.4) (−0.45; 2.39)	0.81
At 5-year follow-up	0.95 (0.75) (−1.5; 2.1)	0.9 (1.05) (−2.35; 2.4)	0.77
F-BIC			
After loading	1.05 (0.75) (−0.75; 2.35)	1.05 (1.1) (−1.08; 2.95)	0.83
At 5-year follow-up	0.50 (0.50) (0.01; 2.48)	0.47 (0.65) (−1.35; 3.4)	0.94

**Table 7:** Membrane perforations according to implant length groups, pre-operative RCBH groups, and perio-groups. For all variables, values are presented as n (%).

VARIABLE	MEMBRANE PERFORATION				*p*-Value
	Yes		No		
	n	%	n	%	
IMPLANT LENGTH					
5 mm	0	0.00	32	100.00	0.10
6 mm	9	9.00	91	91.00	
8 mm	3	13.04	20	86.96	
PRE-OPERATIVE RCBH					
<4 mm	3	7.32	38	92.68	0.21
4–5 mm	8	11.43	62	88.57	
>5 mm	1	2.27	43	97.73	
HISTORY OF PERIODONTAL DISEASE					
No	3	9.38	29	90.63	0.46
Yes	9	7.32	114	92.68	

**Table 8:** Comparison between satisfaction scores given seven days after surgery and given at five-year recall appointment. Unit of comparison was the patient. Values are presented as median (iqr) and (max; min).

	PRE-OPERATIVE	AT 5-YEAR FOLLOW-UP	*p*-Value
SATISFACTION SCORES	8.2 (1.49) (10; 5)	9 (2) (10; 6)	<0.001

**Table 9:** Summary of main findings related to comparisons between the 3-year previously published study [39] and the 5-year present study; main findings of the 3-year intermediate follow-up of the present study were also reported. Even if study samples were different, outcomes regarding implant survival, F-BIC, and general trend of values over time for residual crest and sinus lift were comparable between the two studies, with similar decreasing variations.

	PRE- OPERATIVE	IMPLANT PLACEMENT	PROSTHETIC LOADING	3-YEAR FOLLOW-UP	5-YEAR FOLLOW-UP
n. Implants 3-year study	51	51	51	49	
n. Implants 5-year study	155	155	151	151	147
n. Patients 3-year study	31	31	31	29	
n. Patients 5-year study	79	79	75	75	71
Implant survival 3-year study				96.08%	
Implant survival 5-year study				97.42%	94.84%
Residual crest 3-year study (mm)	5.20 (1.41) (10.66; 2.74)	10.27 (2.15) (15.08; 7.81)	8.88 (2.35) (15.00; 6.09)	7.59(1.97) (14.27; 5.23)	
Residual crest 5-year study (mm)	4.45 (1.3) (0.56; 6.2)	9.25 (2.13) (3.91; 14.55)	6.35 (1.73) (−0.06; 9.87)	5.73 (1.27) (−1.88; 9.85)	5.25 (1.68) (−2.15; 9.46)
Sinus lift 3-year study (mm)		4.84 (1.38) (8.02; 2.17)	3.96 (1.25) (6.33; 1.19)	3.17 (1.13) (6.01; 0.76)	
Sinus lift 5-year study (mm)		4.8 (2.46) (1.9; 9.7)	3.06 (1.3) (0.4; 6.15)	2.53 (0.98) (0.02; 5.05)	1.46 (1.06) (0.03; 4.82)
first bone-to-implant contact 3-year study (mm)			0.26 (0.33) (1.08; −1.34)	0.37 (0.45) (1.92; −0.31)	
First bone-to-implant contact 5-year study (mm)			1 (0.74) (−1.08; 2.95)	0.75 (0.31) (−1.28; 3.01)	0.5 (0.65) (−1.35; 3.4)
Satisfaction 3-year study		8 (2) (10; 5)		9 (1) (10; 7)	
Satisfaction 5-year study		8.2 (1.49) (10; 5)		9 (1) (10; 7)	9 (2) (10; 6)

**Figure A1:**
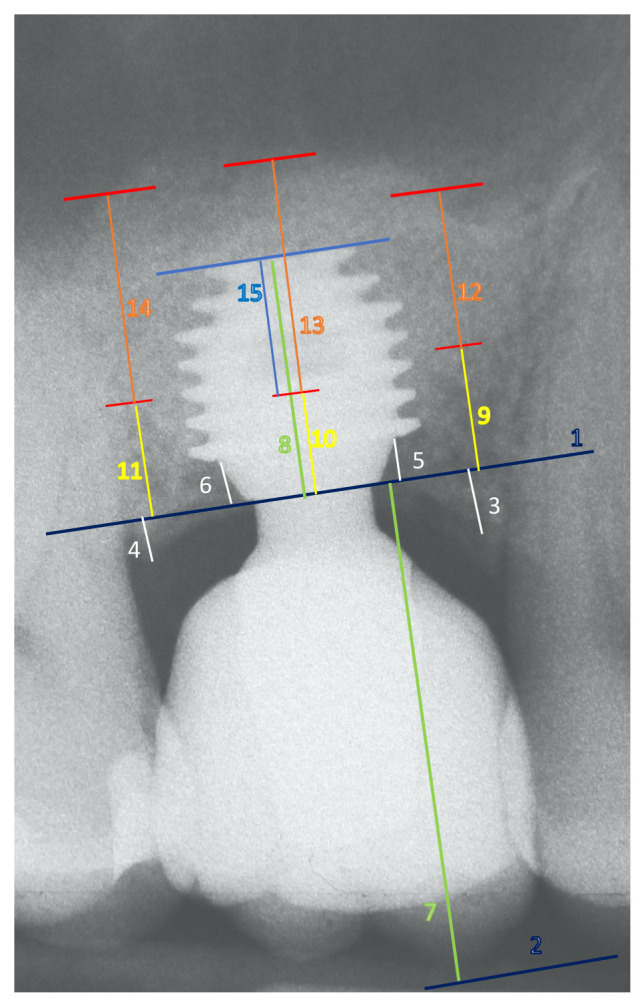
Schematic example of the references for peri-implant bone levels and sinus floor level measurements: (1) Implant–abutment interface; (2) most occlusal point line; (3) crestal bone level (CBL) on the mesial side; (4) CBL on the distal side; (5) first bone-to-implant contact (F- BIC) on the mesial side; (6) F-BIC on the distal side; (7) crown length; (8) implant length; (9) sinus floor level (SFL) on the mesial side; (10) SFL on the central side; (11) SFL on the distal side; (12) sinus lift (SL) on the mesial side; (13) SL on the central side; (14) SL on the distal side; (15) implant protrusion into the sinus (IPS).

## Data Availability

The data presented in this study are available on request from the corresponding author.

## Appendix A

### Appendix A.1. Exclusion Criteria

Exclusion criteria considered [39] were: the presence of active infection at an implant site; ASA status III [42], that is, severe systemic diseases or substantive functional limitations that contraindicated implant surgery (such as drug or alcohol abuse, uncontrolled diabetes mellitus, immunosuppression or immunodepression, severe autoimmune diseases, treatment or past treatment with intravenous amino-bisphosphonates for metastatic bone diseases, radiotherapy to head or neck within two years prior to treatment, history of malignancy or chemotherapy within the previous year, treatment with oral amino-bisphosphonates for more than three years, morbid obesity, active hepatitis, severe renal disease, severe cardiovascular conditions, and recent history of myocardial infarction (MI) or transient ischemic attack (TIA)); ASA status IV, V, and VI [42]; history of sinus surgery; acute or chronic maxillary sinusitis; oro-antral fistulae; untreated periodontitis; poor oral hygiene and motivation; current pregnancy or lactation; heavy smoking (more than 25 cigarettes per day); and severe clenching or bruxism.

### Appendix A.2. Surgical Protocol

Pre-operative assessment consisted of clinical and radiographic evaluation [39]. Panoramic radiographs were used for initial screening, followed by CBCT scans to precisely quantify the amount of available bone under the maxillary sinus. Furthermore, an intraoral radiograph performed with parallel technique was made to determine the baseline RCBH and to allow future comparison with the CBCT scan measurement. When the operative site involved more than one tooth, diagnostic casts for the creation of a mucosal supported surgical guide were made. One month before surgery, each patient underwent a full-mouth session of scaling and root planning using mechanical and hand instrumentation and received personalized oral hygiene instructions.

A pre-operative medication consisting of 2 g of Augmentin (875 mg amoxicillin plus 125 mg clavulanic acid) or 1 g of Klacid (Clarithromycin 500 mg) if allergic to penicillin was given one hour before surgery. All surgical procedures were performed under local anaesthesia, using only Articain 4% with adrenaline 1:100,000 (Citocartin) or Articain 4% with adrenaline 1:100,000 (Citocartin), associated with oral sedation (Halcion 0.25 mg).

After a mid-crestal incision, buccal and palatal full-thickness flaps were reflected. Vertical releasing incisions were made only if necessary. The recipient sites were marked with a 2.0 mm round drill. If the edentulous space involved more than one tooth, the mark was made in accordance with the pre-prepared surgical templates. The osteotomy was initiated using a 2.0 mm diameter pilot drill to a depth of 0.5 to 1.0 mm from the sinus floor while being guided by pre-operative radiographs and the CBCT. The expansion of the osteotomy sites continued with successively larger dedicated manual reamers to create an osteotomy of 5.0 mm diameter and 1.0 mm from the sinus floor. The sinus floor fracture was obtained by inserting a 5.0 mm sinus lift osteotome into the osteotomy to the level of the sinus floor and gently tapping the osteotome with a mallet to create a hairline fracture in the floor of the sinus. Great attention was given to avoid perforation of the sinus membrane. After completion of this procedure, the integrity of the Schneiderian membrane was manually confirmed by gentle sounding with a blunt-tipped depth gauge. The membrane was then elevated by placing a synthetic bone graft material into a syringe and injecting it into the osteotomy. As the column of graft material was advanced in the osteotomy, it gently lifted the sinus membrane to the desired height. Any type of resorbable membrane was not used to protect the Schneiderian membrane before the insertion of the graft material. Implants were placed immediately after the sinus elevation using an implant inserter and using the implant to further raise the sinus floor [39].

Before implant placement, a sinus lift temporary abutment was inserted into the implant to prevent the implant from migrating into the sinus. The flaps were accurately sutured, allowing for a primary wound closure, and all implants were left submerged during the following six-month healing period. Immediately after flap closure, periapical radiographs, which would serve as baseline for future comparison, were made with the paralleling technique [39].

Patients received detailed post-operative instructions, along with antibiotic and analgesic prescriptions. After one week, patients were monitored for evidence of post-operative swelling and/or headaches. The sutures were removed after two weeks, and patients were instructed not to use removable dentures during the six-month healing period [39].

### Appendix A.3. Study Variables and Outcomes

#### Appendix A.3.1. Implant Survival

Implant failure was considered as the need for implant removal either before loading (due to no osseointegration) or after loading (due to excessive bone loss). Implant survival was considered as the implant’s state of being in function at the three-year follow-up evaluation, that is, symptom-free and without mobility, radiolucency, or bone loss so severe as to warrant implant removal.

#### Appendix A.3.2. Peri-Implant Bone Levels and Sinus Floor Level

A secondary outcome included variations of peri-implant bone levels and sinus floor level [39], which were measured through digitally scanned intraoral radiographs and performed with parallel technique using Rinn centring devices (Rinn XCP Posterior Aiming Ring-Yellow, Dentsply, Elgin, IL, USA), immediately after implant placement at healing abutment placement, at prosthetic loading, and after five years of loading. The implant–abutment interface (IAI) was taken as a reference for measurements (Figure A1).

A descriptive analysis of crestal bone level (CBL, average bone level around implants at mesial and distal sides, in mm) and first bone-to-implant contact (F-BIC, in mm), along with their variations ΔCBL (average bone loss) and ΔF-BIC (average apical shift of the first bone-to-implant contact point position), was conducted [39,43]. These values were determined based on changes that took place between loading time and the five-year follow-up time, according to covariates. CBL was measured on mesial and distal sides as the linear distance between the IAI and the highest point of the interproximal bone crest parallel to the lateral sides of the implant body. A positive value was given when the crest was located coronally to the IAI, and a negative value was given when the crest was located apically to the IAI. F-BIC was defined as the first most coronal bone-to-implant relationship visible at the first line of contact on both mesial and distal sides. If F-BIC matched with IAI, the measurement was 0. If it was located apically, the measurement was a positive value. For every implant, an average (av) mesial-distal value (av-CBL and av-FBIC) was calculated at each examination interval.

Furthermore, as described in the literature, implants were divided into two groups on the basis of presenting a crown-to-implant ratio (CIR) less than or greater than 2 [39,48]. The crown height was measured on the radiograph immediately after the prosthetic loading, from the most occlusal point to the IAI. Anatomical CIR (in which the fulcrum is positioned at the interface between the implant shoulder and the crown–abutment complex) was calculated by dividing the digital length of the crown by the digital length of the implant. Measurements [39] were assessed with the aid of a software program (Rasband, W.S., ImageJ, U.S. National Institutes of Health, Bethesda, MD, USA), which uses a measuring tool in conjunction with a magnification tool. To correct the distortion of the radiographic image, the apparent size of each implant (measured directly on the radiograph) was compared with the actual length of the implant to determine with adequate precision the amount of change in the crestal bone around each implant. Setting up the implant length as a known initial reference, the measurements were made to the nearest 0.01 mm. Beyond that, the results from the pre-operative periapical radiographs were compared with those of pre-operative CBCT scans. If disagreements were present between the values, the CBCT values were chosen and served as reference for future comparison with the radiographs.

One dentist, who was not involved in the treatment of the patients, completed all the measurements on periapical radiographs and CBCT scans; the observation intervals of the radiographs were masked to the examiner [39]. Before the start of the study, this investigator was calibrated for adequate intra- and inter- examiner levels of reproducibility in recording the radiographic parameters. The calibration for intra-examiner reproducibility was done with double recording of 25 measurements (25 implants), with an interval of 24 h between the first and second recording. Four basic parameters directly connected to CBL, F-BIC, SFL, and CIR were measured on three radiographs and utilized for this purpose: mesial CBL, mesial F-BIC, mesial SFL, and crown height, all at prosthetic loading. An average value lower than 0.20 mm was considered reliable as threshold limit from a clinically point of view. Furthermore, the abovementioned exercise, according to the same method, was repeated by another dentist (always not involved in the treatments of patients) for inter-examiner reproducibility. In both cases, according to the Bland–Altman method [84], four plots were obtained, with respective average values of difference between each pair of measurements, together with confidence intervals (C.I.).

### Appendix A.4. Statistical Analysis

For data collection, a database including all patients evaluated in the study was created with Microsoft Excel. All data analysis was carried out using Stata v.13.0 for Macintosh (StataCorp, College Station, TX, USA) [39]. The normality assumptions for continuous data were assessed by using the Shapiro–Wilk test; mean and standard deviation (SD) were reported for normally distributed data (mean ± SD), median, and interquartile range (iqr) otherwise (median (iqr)). For categorical data, absolute frequencies, percentages, and 95% confidence intervals were reported. The association between categorical variables was tested with χ2 test; if any of the expected values was less than 5, a Fisher’s exact test was performed. The comparison between the means of continuous variables in two different times was performed by using paired Student’s *t*-test or Wilcoxon matched-pairs signed-rank test. The comparison between the means of two different groups was performed using unpaired Student’s *t*-test, or Wilcoxon rank-sum test. The comparison of the means among more than two groups was done using one-way analysis of variance (ANOVA) or Kruskal–Wallis equality-of-populations rank test as appropriate. Bonferroni correction for multiple comparison was applied. Significance level was set at 0.05.

The study presents compliance with the STROBE checklist guidelines [85].
